# A link between thrifty phenotype and maternal care across two generations of intercrossed mice

**DOI:** 10.1371/journal.pone.0177954

**Published:** 2017-05-19

**Authors:** Bruno Sauce, Carolina P. Goes, Isabela Forti, Bruno Gabriel O. do Monte, Isabela M. Watanabe, Joao Cunha, Andrea C. Peripato

**Affiliations:** 1 Department of Psychology, Program in Behavioral and Systems Neuroscience Rutgers University, Piscataway, United States of America; 2 Institute of Biomedical Sciences, Program in Cellular Biology and Histology Universidade de Sao Paulo, Sao Paulo, Brazil; 3 Department of Genetics and Evolution, Center of Health and Biological Sciences Universidade Federal de Sao Carlos, Sao Carlos, Brazil; University of Missouri Columbia, UNITED STATES

## Abstract

Maternal effects are causal influences from mother to offspring beyond genetic information, and have lifelong consequences for multiple traits. Previously, we reported that mice whose mothers did not nurse properly had low birth weight followed by rapid fat accumulation and disturbed development of some organs. That pattern resembles metabolic syndromes known collectively as the thrifty phenotype, which is believed to be an adaptation to a stressful environment which prepares offspring for reduced nutrient supply. The potential link between maternal care, stress reactivity, and the thrifty phenotype, however, has been poorly explored in the human and animal literature: only a couple of studies even mention (much less, test) these concepts under a cohesive framework. Here, we explored this link using mice of the parental inbred strains SM/J and LG/J–who differ dramatically in their maternal care–and the intercrossed generations F1 and F2. We measured individual differences in 15 phenotypes and used structural equation modeling to test our hypotheses. We found a remarkable relationship between thrifty phenotype and lower quality of maternal behaviors, including nest building, pup retrieval, grooming/licking, and nursing. To our knowledge, this is the first study to show, in any mammal, a clear connection between the natural variation in thrifty phenotype and maternal care. Both traits in the mother also had a substantial effect on survival rate in the F3 offspring. To our surprise, however, stress reactivity seemed to play no role in our models. Furthermore, the strain of maternal grandmother, but not of paternal grandmother, affected the variation of maternal care in F2 mice, and this effect was mediated by thrifty phenotype in F2. Since F1 animals were all genetically identical, this finding suggests that maternal effects pass down both maternal care and thrifty phenotype in these mice across generations via epigenetic transmission.

## Introduction

Maternal effects can be defined as the causal influences from mother to offspring beyond genetic information [1]. The variation in maternal performance, such as nursing [2], grooming/licking [3], and nest building [4], have lifelong consequences on offspring development [5–9]. If a set of maternal effects alters the future maternal performance of the offspring, then the set can become semi-permanent in the female line–from mothers to daughters to grand-daughters, and so on [10]. In rodents, for example, the quality of maternal licking/grooming can be transmitted across three generations of fostered rats, and is associated with methylated genes in the brain [11].

Environmental changes experienced by mothers may also have an impact not only on offspring’s maternal performance, but also on their physiology. In humans, an interesting case in the Netherlands during World War II revealed that females exposed to famine in utero were more likely to have, later in life, high glucose levels, hypertension, and coronary heart disease [12]. The grandchildren of those women who gestated during the famine had high rates of fat accumulation during birth and poorer health overall [12]. This pattern of fat accumulation and disturbed growth pattern under food deprivation at birth can be defined as “thrifty phenotype.” The thrifty phenotype seems to be caused by changes in the expression of specific genes due to cell structure changes, oxidative stress, and epigenetic markers [13,14], and it has been seen also in animals [15]. In rodents, adverse conditions such as scarcity of food directly affect the emotional state of the mother and, thus, quality of maternal care [16]. Under such conditions, a mother in a highly demanding environment should transmit to their young an enhanced level of stress reactivity in anticipation of significant environmental adversity. Therefore, the preparation of the offspring could lead not only to the growth/metabolic changes of the thrifty phenotype, but also to greater stress reactivity in general. If either or both of these changes lead to poor maternal care, this pattern could repeat itself through generations.

In previous research, we found that mice from an intercross between two inbred strains, SM/J and LG/J, whose mothers did not nurse properly, had low birth weight early in development followed by rapid weight gain and irregularly concentrated fat in central areas like the viscera [17]: patterns that match the thrifty phenotype. We also investigated the maternal performance of females and we identified genomic regions (QTLs) related to offspring survival [18], litter size [19], stress reactivity/emotionality (unpublished data), nest building [20], and nursing [17]. Furthermore, differences in maternal care between the two inbred strains were associated in the brain with different levels of mRNA expression of an epigenetically controlled gene, *Paternally-expressed gene 3* (*Peg3*) [21], suggesting that the maternal performance we observed may be passed down through generations. The potential link between maternal care, stress, and the thrifty phenotype has been poorly explored, and only a couple of studies even mention (much less, test) these concepts under a cohesive framework. In the current study, we explored this link using the parental inbred strains SM/J and LG/J, and the two generations of intercrossed F1 and F2 mice. These mice offer great potential because the genetically identical individuals (F1) were raised by either remarkably good mothers (SM/J) or remarkably bad mothers (LG/J). We measured the maternal care, the thrifty phenotype, and the stress reactivity of animals of the F2 generation and related those traits to the type of grandparents they had. Since F1 are all identical, differences in the information passed down from grandmothers (SM/J or LG/J) to F2 should reflect maternal and epigenetic effects, as opposed to genetic ones.

**Fig 1:**
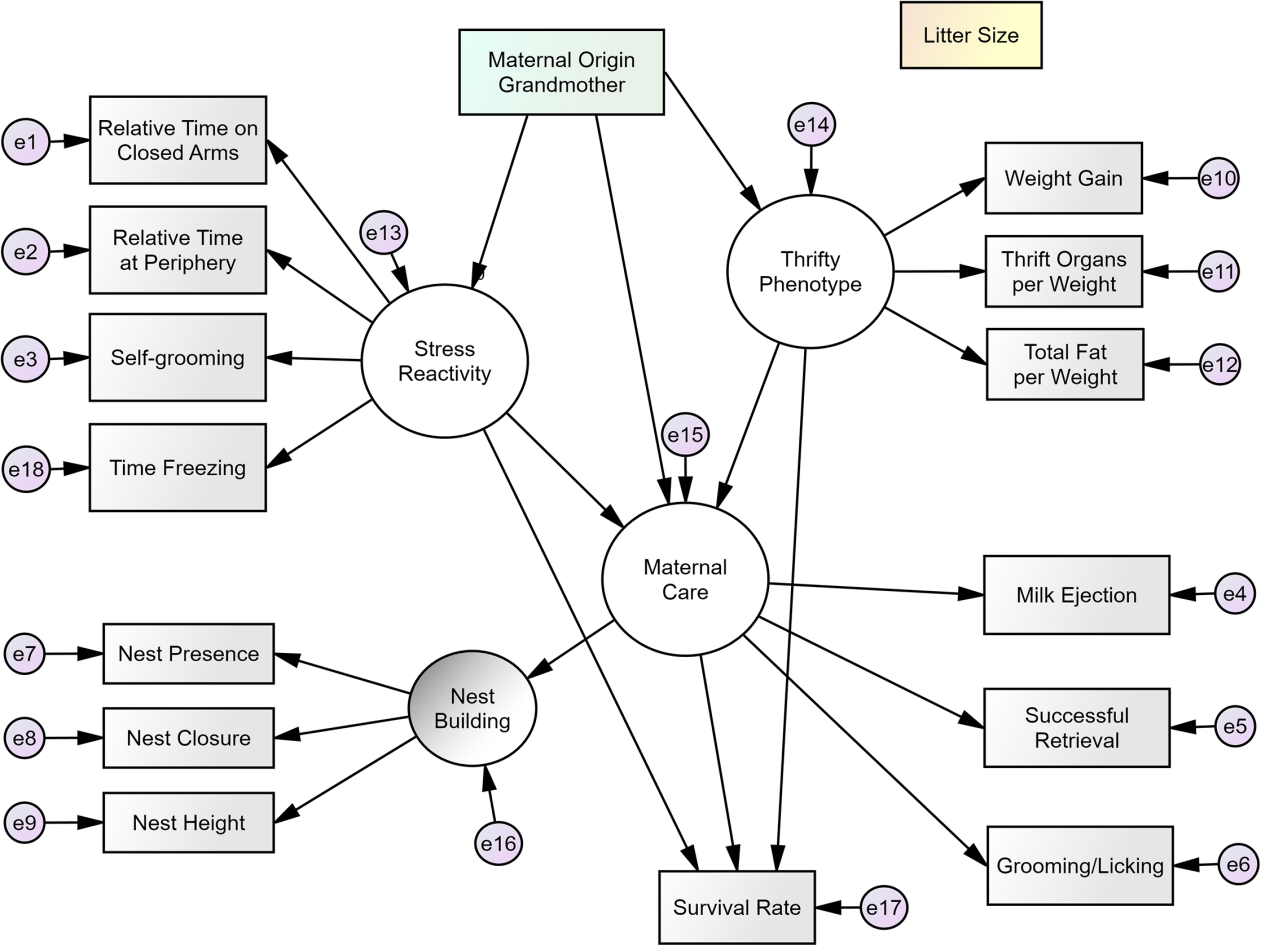
Proposed model of the relationship between maternal origin, stress reactivity, thrifty phenotype, maternal care, and offspring survival. Litter size is a covariate in the model. Small circles with an “e” represent error (leftover variance due to flaws in measurements and estimations). Arrows going out of the latent factors (big circles) represent factor loadings, while all other arrows represent regression weights. The model overall had a bad fit to the data. After testing the measurement model with all variables here, we revised this proposed model.

We used correlational approaches in this study, as these can complement past findings with experimental approaches and help answer some relevant questions on the topic (for a discussion on these two different approaches in behavioral sciences, see [22]). Evidence for the role of maternal effects in mammals usually comes from studies that deprive pups from their mothers for long periods of time, or that disturb the pregnancy, or that use cross fostering designs. These experimental studies are all useful and provide great insight on the topic. However, they are ill suited to answer questions such as how maternal care contributes to changes in offspring under relatively normal conditions, or the extent to which variation in a trait is connected to variation in maternal performance in a population. Thus, studying naturally occurring individual differences within a group of animals allowed us to examine the influences of stress reactivity and thrifty phenotype on maternal behaviors in the way they might be related in a wild population (as expected if these traits evolved together). Another issue with the literature on maternal effects is the lack of statistical models examining multiple relationships between phenotypes simultaneously. These relationships can go undetected if one is using simpler techniques such as Multivariate Analysis of Variance (MANOVA) or multiple regression analysis. For more on this, see [23]. Here, we used confirmatory factor analysis with our measurements to verify and derive the latent phenotypes of stress reactivity, maternal care, and thrifty phenotype, since by definition, none of those three phenotypes can be measured by a single criterion. From these latent phenotypes, we then used structural equation modeling to test their potential relationships under our main model (Fig 1), and compared it to some alternative models. This main model is derived from our belief based on the evidence summarized above: Given that the strain of the grandmother (SM/J or LG/J) differ dramatically in their maternal care, this would later influence the variation in the granddaughters’ (F2) own maternal care, as well as the variation in their stress reactivity and levels of thrifty phenotype. In addition, the main model hypothesizes that stress reactivity and thrifty would inherently covary with maternal care in the granddaughters, and that each of these three phenotypes influences the survival rate in the offspring (F3).

## Materials and methods

### Animals and breeding

We used inbred mouse strains Small (SM/J) and Large (LG/J), and their F1 and F2 intercrosses. The two strains were acquired from Jackson Laboratory (Bar Harbor, ME, USA). The SM/J strain came from a selection experiment for small body size at 60 days while the LG/J strain originated separately from an albino population selected for large body size at 60 days (for history and details, see [24]). For our study, we used the second generation of each strain, which was bred in our facilities to guarantee that our starting groups experienced similar environmental conditions as the later intercrossed generations. The starting groups (called here the “grandparents”) consisted of 59 SM/J animals (31 females and 28 males) and 46 LG/J animals (20 females and 26 males). We mated 10 SM/J males with LG/J females and 10 LG/J males with SM/J females, resulting in the F1 generation (34 F1 females and 29 F1 males). We then performed crosses among F1 mice in all combinations of origin (F1 female from SM/J mother mated with F1 male from LG/J mother, F1 female from LG/J mother mated with F1 male from LG/J mother, etc.), and obtained 242 F2 females and 222 F2 males. Finally, we randomly mated the F2 mice in order to have F2 mothers expressing maternal phenotypes.

All animals were fed *ad libitum* with Nuvilab CR1/Nuvital (Colombo, Brazil) and maintained at a constant temperature of 21°C under a 12-hour light/dark cycle at Universidade Federal de Sao Carlos. At 21 days of age we weaned all pups and placed them in single-sex cages with at most five animals. On their seventh week, we mated the animals in a paired mating system. Males were removed from the breeding cage when pregnancy was detected by an increase of the female’s abdominal circumference and weight gain. After birth, each female stayed with their offspring until weaning. We performed all measurements and behavioral tests during the same period (8:00 a.m. to 12:00 p.m.) in the same colony room. This study was carried out in accordance with the Guidelines for the Care and Use of Mammals in Neuroscience and Behavioral Research (ILAR, USA). The protocol was approved by the Ethics Committee of the Universidade Federal de Sao Carlos (CCEA, Brazil. Permit Number: 018/2006). Animals were euthanized using the method of cervical dislocation.

### Latent and measured phenotypes

The inbred strains SM/J and LG/J show dramatic differences in maternal care [18,25], and the F1 mice, although genetically identical (with the exception of mitochondrial DNA), were cared for by either “bad mothers” (females LG/J) or “good mothers” (females SM/J). We used the variable of “Maternal Grandmother” (two categories: either F1 mothers cared by SM/J mothers or F1 mother cared by LG/J mothers) for each F2 mouse as the independent/exogenous variable in our main model, as shown in Fig 1. Because we performed crosses among F1 mice in all combinations of origin, mice from the F2 generation came from male and female F1 animals whose grandmothers were either SM/J or LG/J. This balancing allowed us to test potential epigenetic effects that are not due to maternal effects. For example, it is possible that F2 animals with SM/J grandmothers are good mothers not because of maternal effects passed down the maternal line (female F1), but because the SM/J might have imprinted genes that passed down in the paternal line (male F1). We used the variable of “Paternal Grandmother” (two categories: either F1 fathers cared by SM/J mothers or F1 fathers cared by LG/J mothers) to test this alternative model. All other phenotypes in the current study were derived from measures in F2, and consisted of dependent/endogenous variables.

We measured a total of 15 different phenotypes in F2 females, and most of these measure the main latent phenotypes of maternal care, stress reactivity, and the metabolic disorder known as the thrifty phenotype (hereinafter referred to simply as “thrifty” or “thrifty phenotype”). For the latent phenotype of maternal care, we measured nest building (nest presence, nest closure, and nest height), successful pup retrieval, grooming/licking, and nursing. For the latent phenotype of stress reactivity, we measured self-grooming, and the behaviors from the tests of Open Field, Elevated Plus Maze, and Forced Swim. None of these tests are direct/pure measures of stress, but we believe they should capture some component of stress reactivity, whether as a trigger to anxiety, repetitive behavior, exploration, or depression. For the latent phenotype of thrifty, we measured relative fat (as the percentage of total body weight), relative weight (also as the percentage of total body weight) of organs related to the thrifty phenotype (such as heart, liver, and reproductive apparatus), and weight gain since birth. In order to test an evolutionarily meaningful outcome of the three main latent phenotypes, we measured the rate of offspring survival from each of the F2 mothers. In addition, since litter size is a potential confounding factor in our measurements, we measured litter size from each F2 mother and used this value as a covariate in our analyses.

Regarding sample size, there were 242 F2 female mice in total for the latent phenotypes of maternal care, stress reactivity, and thrifty. For structural equation modeling, the suggested minimum sample size varies in the literature, but our sample seems to be adequate; it is considered a medium sample for such analyses [23]. Furthermore, using the power analysis described in [26], our sample size of 242 allows our main model containing 93 degrees of freedom (Fig 1) to be tested with the excellent statistical power of 0.98.

### Procedures for the measured phenotypes

The procedures used for measuring the phenotypes are described below according to their respective main latent phenotype. We recorded all tests with a video camera, and a single person evaluated all females for each test.

#### Maternal care

Nest Building: We measured nest building behavior as described in [20]. We evaluated prepartum nest building in F2 females daily until delivery, through the variables of Nest Presence (0 = absent; 1 = present), Nest Closure (0 = opened; 1 = closed), and Nest Height (centimeters).

**Pup retrieval:** Females usually perform protective behavior and keep their offspring inside the nest by bringing their pups back when they drift outside or are removed by another animal [27]. We observed the behavior of pup retrieval on the first, third, and seventh day postpartum after removing all pups from their mother and putting them back to the cage in random locations. We recorded this for 6 min, and measured the relative number of pups successfully returned to the nest. Previous results indicated that six minutes were enough to record pup retrieval and other related behaviors, and poor performance here means that pups were left spread out through the cage. It is also worth mentioning that extraneous odors could confuse the pup retrieval behavior. To avoid this issue, we handled pups inside a laminar flow, always used gloves, and cleaned surfaces outside the cage with 70% ethanol.

**Grooming/Licking:** During the recordings of the procedure for pup retrieval, as described above, we also measured the time each mother spent grooming and licking her offspring. We observed grooming and licking on the first, third, and seventh day postpartum. This maternal behavior is typical in rodents, with substantial individual variation during the first week postpartum [28].

**Nursing:** We measured nursing indirectly by detecting the presence of milk in the stomach of pups from F2 mothers during the first day after delivery, as described in [17]. This phenotype was measured as a binary variable (i.e., a categorical variable that can only take two values): 1 represents the presence of milk and 0 represents its absence. Generally, females with milk ejection at D1 maintained this behavior during the seven days evaluated.

#### Stress reactivity

Open Field: The Open Field test is commonly used test for anxiety, in which mice are allowed to explore a novel and typically stress-inducing open space (for a review on the topic, see [29]). Here, we tested the mothers on the second day postpartum. The apparatus consisted of a round arena (30 cm in diameter) with acrylic transparent walls (30 cm high). Females were transported with their pups to a test room that had dim lights and mild noise intensity. Then, we removed the mothers from their cages and immediately placed them in the center of the arena with their heads facing the same side. We recorded their movements for 5 minutes with a video camera. We quantified each female’s relative time spent in the center of the arena, and used this as an indirect measure of stress reactivity (via the manifestation of anxiety-like behaviors), in which less time spent exploring the center of the arena indicates more stress [30].

**Elevated Plus Maze:** The Elevated Plus-Maze is another commonly used test of anxiety, similar to the Open Field test, in which mice are allowed to explore stress-inducing open spaces (for a review, see [31]). We tested the mothers on the fourth day after delivery. The cross-shaped apparatus consists of two open arms (30 × 5 × 0.25 cm) and two closed arms with acrylic transparent walls (30×5×15 cm). The cross fit into a base raised 38.5 cm above the floor. We transported the mothers in the same way that we did for Open Field, then placed each female in the center of the cross with her head facing an open arm. We recorded their movements for 5 min with a video camera. The test room had dim lights and mild noise intensity. We quantified the relative time each female spent in the open arms as an indirect measure of stress reactivity, such that less time indicates more stress. We considered females to have moved when all four paws reached a different area.

**Forced Swim:** The Forced Swim is a commonly used test of depression that measures a mouse’s behavior during extreme stress. This test is based on the principle that some mice stop swimming when exposed to an inescapable situation (for a review on the topic, see [32]). Given that depression is commonly reactive in nature, stress is known to play a more than trivial role in many cases. On the sixth day after delivery, we placed each female in a glass cylinder (40-cm deep by 20 cm in diameter) filled with water (19.5 cm high) at 24°C, and recorded their movements for 6 min. Evidence on the Forced Swim in mice (though not in rats), shows that one short day of testing is sufficient for stable and reliable results [32]. We recorded the amount of time animals spent immobile during the final 4 min of the test (since the initial two minutes are prone to interference), and more time spent immobile indicates higher stress reactivity.

**Self-grooming:** During the recordings of the procedure for pup retrieval (described above), we also measured the time that each mother spent self-grooming. We observed self-grooming on the first, third, and seventh day postpartum. High levels of self-grooming indicate elevated stress reactivity [33].

The stress reactivity tests used here were ordered from least invasive (Open Field) first to most stressful (Forced Swim so as to minimize stress carried over between tests. All females were tested in the same way for consistency.

#### Thrifty phenotype

In thrifty phenotypes, low birth weight is usually associated with lower adult body size, lean mass and altered fat distribution [34]. In the pattern of catch-up growth found in thrift, the onset of insulin resistance appears to be dependent on high weight gain and is associated with the emergence of central adiposity [35,36]. Lower lean mass is believed to be a key component of thrift, as well as associations between birth weight and later fat mass or fat distribution, when adjusted for current weight [34].

We weighed the F2 females daily during their first week of life, then again on day 21, which was their day of weaning, and then weekly for seven weeks. After the animals were euthanized, we weighed the parts most commonly related to thrifty phenotype: reproductive organs, heart and liver–as well as their reproductive, mesenteric, inguinal, and kidney fat pads.

### Statistical analyses

For all measured phenotypes, we defined univariate outliers as any values above or below two interquartile ranges, and changed them to values at either the lower quartile for low outliers, or upper quartile for high outliers (for the advantages of this technique, popularly known as “bring it to the fence”, over the deletion of data, see [37]). After treating univariate outliers, we checked for the existence of multivariate outliers using the Mahalanobis distance. We also tested all measured phenotypes for the presence of kurtosis and skewness, and when necessary, performed relevant transformations to fit the variables to a normal distribution. These pre-analyses were all performed in SPSS 21. Regarding missing data, we estimated values for each case by using full information maximum likelihood (FIML) [38] in AMOS 21. This estimation provides less biased information than ad hoc procedures such as listwise deletion, pairwise deletion or imputation of means [39].

In order to explore and test the theoretical model proposed here, we used structural equation modeling (SEM). As a hybrid of multiple regression and factor analysis techniques, SEM allows simultaneous assessment of the strength and direction of the interrelationships among multiple dependent and independent variables, and examines the direct and indirect effects of one variable upon another [23]. Since a structural equation model can have multiple indicators for a single (latent) variable, this reduces measurement error because only the shared variance between measures are considered, leading to more accurate and often stronger relationships between latent variables than is found from other multivariate methods such as MANOVA or multiple regression [23].

We followed a two-step approach recommended by Kline [23], in which a measurement model is examined before the structural model. For the measurement model, we conducted confirmatory factor analyses to determine if the latent variables of maternal care, stress reactivity, and thrift fit our data well. For the structural model, we performed SEM in order to test our main model of connections between the latent variables (Fig 1). The model states that the maternal effects received by F2 from F1 and the parental strains (expressed here as the “Maternal Grandmother”) influenced their maternal care, stress reactivity, and thrifty phenotype, and those effects on stress reactivity and thrifty phenotype, then indirectly influenced maternal care in these F2 females. Further, the model also states that these three latent phenotypes in F2 influenced offspring survival of the next generation (F3). Thus, this model indirectly assesses the transmission of information across generations through maternal effects.

We used the maximum likelihood estimation in AMOS 21 to acquire the solution for our main model (Fig 1). This particular estimation is considered robust in comparison to other procedures like Generalized Least Squares and Asymptotically Distribution-Free, and allows reliable fit indices with relatively small samples [40]. The main model is recursive, and thus it is identified. We assessed model fit by using two absolute indices–Model Chi-Square and Root Mean Square Error of Approximation (RMSEA)–that describe how the model represents the observed data, and for which lower values indicate better fit. For the Model Chi-Square (χ^2^_M_), the null hypothesis is the model itself, so failing to reject it (i.e., a small Model Chi-Square) indicates a good fit (with alpha here set at 0.05) [23]. Similarly, RMSEA values of 0.06 and below are considered good [41]. In addition to these two absolute indices, we also assessed model fit with two incremental indices–Tucker-Lewis Index (TLI) and Comparative Fit Index (CFI). These describe how well the model fits in comparison to a baseline model in which all variables are uncorrelated and without latent variables, and for which higher values indicate better fit [23]. TLI and CFI indicate an adequate model fit at values of 0.95 or above [41]. We chose these tests due to their statistical relevance and frequent use [23,42,43]. For assessing the significance of individual parameters such as regression paths and correlations, we chose an alpha value of 0.05.

We also tested for indirect (mediation) effects in the structural model between stress and thrifty phenotype using the PRODCLIN program developed by MacKinnon, Fritz, Williams, & Lockwood [44]. PRODCLIN examines the product of the unstandardized path coefficients divided by the pooled standard error of the path coefficients (αβ/σαβ) and generates a confidence interval. If the values between the upper and lower confidence limits include zero, this suggests the absence of a statistically significant mediation effect. The PRODCLIN algorithm tests mediational effects without some of the problems inherent to other methods, such as inflated rates of Type I error, and is considered especially suitable for use with SEM [45].

With our main model estimated and tested, we then compared it to other models. To verify the influence of epigenetic or other effects from grandmothers of the paternal line (which, by definition, are not considered maternal effects), we used a model with the variable of “Paternal Grandmother” (F1 fathers cared by SM/J mothers or F1 fathers cared by LG/J mothers) instead of “Maternal Grandmother”. Since this is a non-hierarchical comparison, we compared the values between models for the fit index of AIC, and considered a difference of more than 10 scores to indicate superiority of the better fitted model [23]. In addition, we compared our main model to models with different relationships between the variable of “Maternal Grandmother” and the three main latent phenotypes (maternal care, stress reactivity, and thrift) in F2. For hierarchical comparisons between models, we performed the Chi-Square Difference Test (χ^2^_D_), where the null hypothesis represents no differences between the models [23].

## Results

### Descriptive statistics

The means and standard deviations for all measured variables are shown in Table 1, while inter-correlations between all variables are shown in Table 2. By examining all 15 variables for the presence of univariate outliers (any value above or below two interquartile ranges), we found up to seven cases of outliers in five variables: Nest Height, Grooming/Licking, Relative Time on Closed Arms, Relative Time on Periphery, and Time Freezing. Instead of deleting these cases from the analyses, however, we “brought them to the fence” by setting their values to the two interquartile ranges (above the 3^rd^ quartile or below the 1^st^ quartile, depending on the value). After treating these outliers, we examined the existence of multivariate outliers using the Mahalanobis distance, and found one case (individual #894). We excluded this mouse from the analyses.

**Table 1:** Means and standard deviations of all measured variables before transformations, with the respective latent phenotype used in the models.

Latent phenotype	Measured variable	Mean	Standard Deviation
*Litter size*	Litter Size (number of pups)	8.48	2.91
*Survival Rate*	Survival Rate (% of total pups)	86.02	31.97
*Maternal Care*	Nest Building: Nest Presence (0 = absent; 1 = present)	0.87	0.22
	Nest Building: Nest Closure (0 = opened; 1 = closed)	0.14	0.22
	Nest Building: Nest Height (centimeters)	3.66	0.96
	Nursing: Milk Ejection (0 = absent; 1 = present)	0.93	0.25
	Pup Retrieval: Successful Retrieval (% of total pups)	45.89	35.31
	Grooming/Licking (seconds)	25.15	16.36
*Stress Reactivity*	Elevated Plus Maze: Relative Time on Closed Arms (%)	82.26	16.94
	Open Field: Relative Time at Periphery (%)	81.85	12.24
	Forced Swim: Time Freezing (seconds)	68.77	68.29
	Self-grooming (seconds)	6.91	7.00
*Thrifty Phenotype*	Weight Gain (grams)	5.04	1.42
	Thrift Organs per Weight (% of total body weight)	17.76	5.39
	Total Fat per Weight (% of total body weight)	7.55	3.63

**Table 2:** Inter-correlations of all measured variables.

	1	2	3	4	5	6	7	8	9	10	11	12	13	14	15
1. Litter Size	—														
2. Survival Rate	.28^**^	—													
3. Nest Presence	.15^*^	.21^**^	—												
4. Nest Closure	-.09	.08	.29^**^	—											
5. Nest Height	.16^*^	.14^*^	.47^**^	.51^**^	—										
6. Milk Ejection^$^	.20^**^	.44^**^	.09	.07	.13	—									
7. Successful Retrieval	-.08	.15^*^	-.04	-.02	.02	.16^*^	—								
8. Grooming/Licking	.01	.11	-.02	.10	.06	.09	.04	—							
9. Relative Time on Closed Arms	-.01	-.06	.06	.16^*^	.14	.08	-.06	.04	—						
10. Relative Time at Periphery	-.11	.09	.04	.14	.00	.10	.06	.05	.23^**^	—					
11. Time Freezing	.10	.00	-.05	.00	-.02	.02	-.03	.07	-.04	.00	—				
12. Self-grooming	-.12	.07	-.01	.02	-.01	.12	-.10	.05	-.30^**^	-.12	-.04	—			
13. Weight Gain	-.04	.05	-.01	-.05	-.15	-.11	-.08	-.09	-.02	.10	.12	.10	—		
14. Thrift Organs per Weight	.04	-.11	.00	.04	-.02	-.02	-.07	.00	.11	-.09	.02	.04	-.16^*^	—	
15. Total Fat per Weight	.03	.00	.04	-.18^**^	-.01	-.24^**^	-.10	-.12	-.06	-.07	.11	-.15	.32^**^	-.12	—

* p < 0.05

** p < 0.01

^$^ Point-biserial correlation performed due to variable’s binary nature

Because SEM can be sensitive to non-normal distributions as with parametric analysis), we tested all variables for skewness and kurtosis. Survival Rate, Nest Presence, and Milk Ejection had significantly negative skewness. In addition, Survival Rate, Nest Presence, Nest Closure, Nest Height, and Milk Ejection had significantly positive kurtosis. We applied a square root transformation to the variable Nest Closure, a logarithmic transformation to the variable Survival Rate (since a square root transformation did not solve the issue of non-normality), and an inverse transformation to the variable Nest Presence (since neither square root nor logarithmic transformations solved the issue of non-normality). These transformations substantially improved the new values for skewness and kurtosis, bringing them below the expected values for normal distributions. Because Milk Ejection is a binary variable, we did not apply any transformations. The data were missing at random, and less than 15% of the whole sample was missing. We estimated values for each missing case by using full information maximum likelihood estimation (FIML) [38], and used these for all further analyses.

### Measurement model

**Fig 2:**
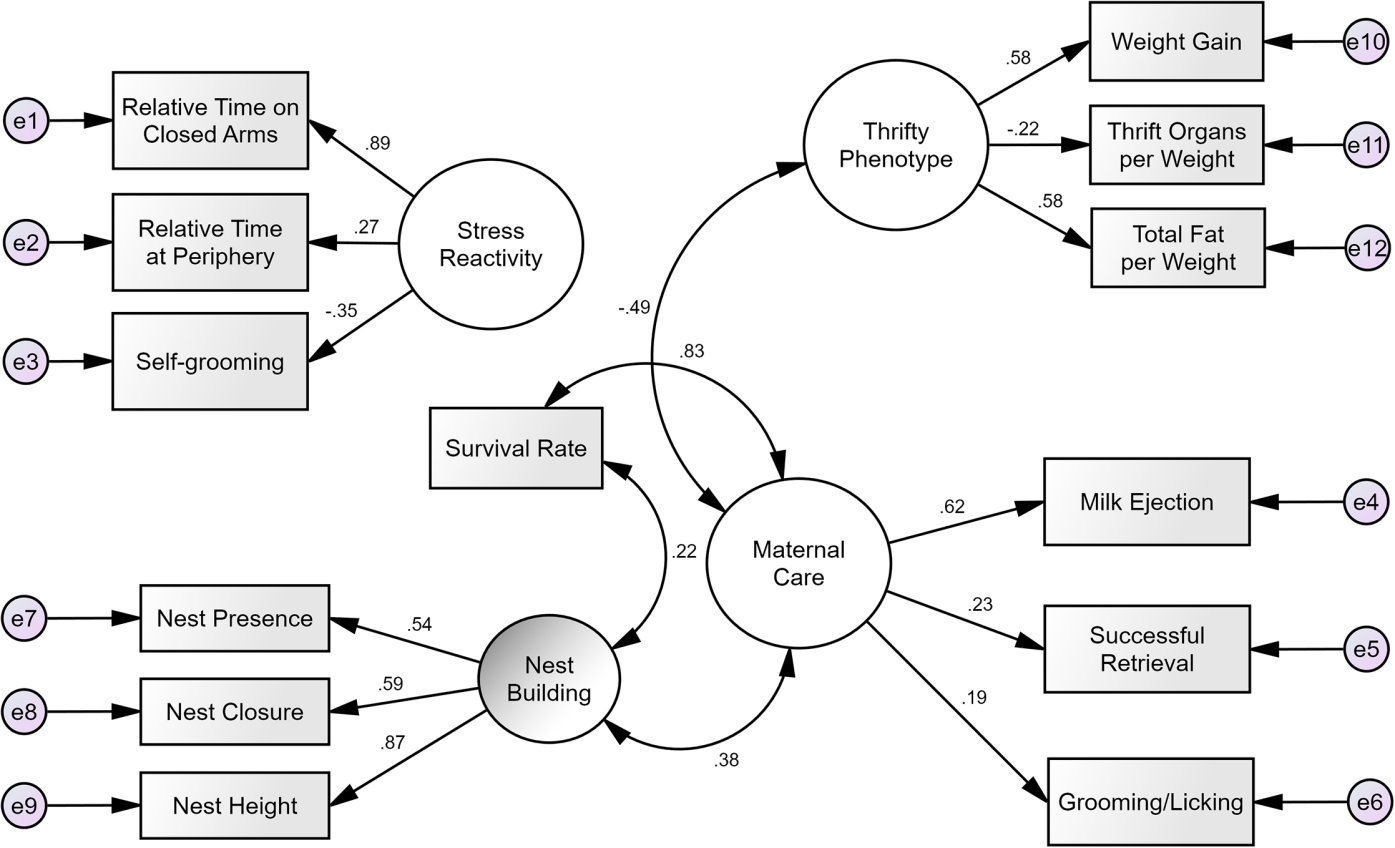
Revised measurement model of all latent phenotypes and offspring survival rate. When testing the model, all latent variables and the measured variable of Survival Rate were set as being potentially correlated. The figure, however, shows only the statistically significant correlations. The model overall had an excellent fit to the data. Small circles with an “e” represent error (i.e., leftover variance due to flaws in measurements and estimations). Arrows going out of the latent factors (big circles) represent factor loadings, while the curved arrows represent correlation coefficients. All parameters shown are standardized.

Since the models in this study comprise three latent phenotypes (Maternal Care, Stress Reactivity, and Thrifty Phenotype), we first examined a measurement model to ensure that the relevant measured variables would form coherent latent variables (as recommended by Kline [23]). In other words: The measurement model is an agnostic model that tests how the variables are related in the absence of any hypothesized relationships, while still capturing only the common variance between the variables. This contrasts with techniques such as principal component analyses, which capture both shared and non-shared variance, and are better suited for purposes of dimension reduction, [23]. For this, we conducted confirmatory factor analyses with the three main latent phenotypes correlated, as well as the latent variable of Nest Building (used in the structure model as a measure of Maternal Care), and the measured variables of Survival Rate and Litter Size as covariates. This first measurement model showed inadequate fit to the data (χ^2^ = 97.0, df = 77, p = 0.061; RMSEA = 0.033; TLI = 0.878; CFI = 0.922), and we discovered that the covariate Litter Size (but not any other latent variable or covariate) was responsible for this issue. From the results of a multiple regression, we found that there were significant effects of differences in litter size only for Nest Presence and Weight Gain, but no significant effects on the other variables. Removing Litter Size from the measurement model then produced an excellent fit to the data (χ^2^ = 66.37, df = 68, p = 0.533; RMSEA < 0.001; TLI = 1.012; CFI = 1.000). However, the variable Time Freezing had, unexpectedly, a non-significant factor loading of -0.01 (p = 0.891) on Stress Reactivity. Due to this, we removed Time Freezing from further analyses. After these two modifications, the final measurement model (Fig 2) had an excellent fit to the data (χ^2^ = 59.2, df = 56, p = 0.361; RMSEA = 0.015; TLI = 0.977; CFI = 0.986), with all measured variables having significant factor loadings (p < 0.05) that ranged between 0.20 and 0.89. The excellent fit of the model and the significant loadings suggest that the empirical measurement of the latent phenotypes is consistent with what we expected from theory. Regarding the latent variables, there were significant correlations between Maternal Care and Thrifty Phenotype (r = -0.490, p = 0.003); Maternal Care and Nest Building (r = 0.376, p = 0.003); Survival Rate and Maternal Care (r = 0.601, p < 0.001); and Survival Rate and Nest Building (r = 0.161, p = 0.003).

### Structural model

After the modifications informed by confirmatory factor analysis of the measurement model, the SEM of our main model had a statistical power of 0.93 (from the original 0.97 before modifications). This represents an excellent power because it greatly exceeds the recommended value of 0.8 for SEM analyses [26].

**Fig 3:**
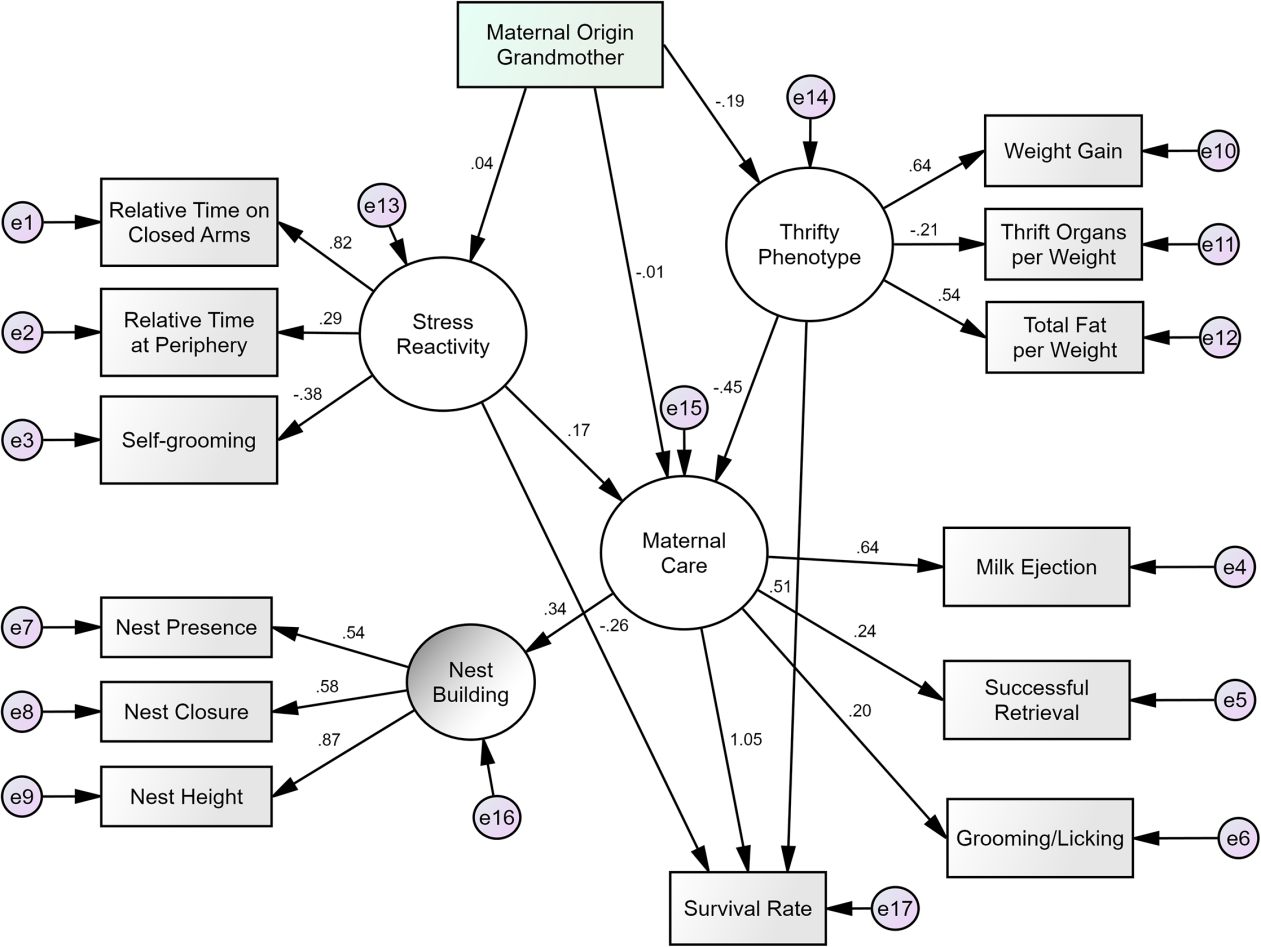
Revised model of the relationship between maternal origin, stress reactivity, thrifty phenotype, maternal care, and offspring survival. The model overall had an excellent fit to the data. Small circles with an “e” represent error (i.e., leftover variance due to flaws in measurements and estimations). Arrows going out of the latent factors (big circles) represent factor loadings, while all other arrows represent regression weights. All parameters shown are standardized.

Structural Equation Modeling analysis of our main model indicated that the model had an excellent fit to the data (χ^2^ = 70.3, df = 70, p = 0.469; RMSEA = 0.004; TLI = 0.998; CFI = 0.999). The factor loadings of each latent phenotype (white circles) on its corresponding observed variables, as well as the regression paths between latent variables can be seen in Fig 3. The path from Thrifty Phenotype to Maternal Care was significant (β = -0.45, p = 0.011), as were the paths from Maternal Care to Survival Rate (β = 1.05, p = 0.001) and from Thrifty Phenotype to Survival Rate (β = 0.51, p = 0.038). It is also important that the path from Maternal Grandmother to Thrifty Phenotype reached borderline significance (β = -0.19, p = 0.052). Contrary to what we expected, however, the paths from Maternal Grandmother to both Stress Reactivity and Maternal Care were not significant. Also, surprisingly, the path from Stress Reactivity to Maternal Care was not significant.

Given the significant path from Thrifty Phenotype to Maternal Care, there could be a partial mediation effect of Maternal Grandmother on Maternal Care via Thrifty Phenotype. By using the PRODCLIN program to test for mediation, we found that the results yielded a lower and upper 95% confidence limit that did not include zero. This suggests a mediation effect linking the type of maternal grandmother (the SM/J strain of good mothers vs. the LG/J strain of bad mothers) to the quality of maternal care in the F2 generation.

To verify the importance of the maternal origin in our main model in contrast to the paternal line, we tested a model where paternal line (defined as either the grandmother SM/J or the grandmother LG/J, similarly to maternal line) takes the place of maternal origin. In other words, in order to conclude that the phenotypes in F2 females were influenced by maternal effects, those effects must have come from the F2 females’ maternal grandmothers, and not from their paternal grandmothers. Indeed, this seems to have been the case. The paternal model had a poor fit to the data (χ^2^ = 85.43, df = 70, p = 0.101; RMSEA = 0.03; TLI = 0.904; CFI = 0.936), and our main model provided significantly better fit in comparison (AIC diff = 15.2, which is higher than the threshold of 10).

To assess the effects of Maternal Grandmother on the latent variables, we further tested our main model against two other possibilities: A1) a model in which Thrifty Phenotype is not influenced by Maternal Grandmother, and does not influence Maternal Care or Survival Rate; and A2) a model in which Stress Reactivity is not influenced by Maternal Grandmother, and does not influence Maternal Care or Survival Rate. Because the A1 model can be considered as nested within our proposed model, we used the Chi-square difference test to see the A1 model could explain the data more parsimoniously. The A1 model was significantly worse than our proposed model, χ^2^ (3, N = 241) = 16.2, p = 0.001. This means that the additional parameters in our proposed model (the relationships with Thrifty Phenotype) are indeed important. The A2 model, however, was not statistically different from our proposed model, χ^2^ (3, N = 241) = 5, p = 0.172. Because the A2 model is more parsimonious (less parameters) than our proposed model, the non significance suggests that Stress Reactivity as measured in this study is not relevant to the overall model.

## Discussion

Here we found a remarkable relationship between the metabolic disorders known as the thrifty phenotype (increased weight gain, high relative amount of fat, and reduced relative weight of thrift organs) and lower quality of critical maternal behaviors, including nest building, pup retrieval, grooming/licking, and nursing. To our knowledge, this is the first study to show, in any mammal, a clear connection between the natural variation in thrifty phenotype and maternal care. Additionally, both traits in our study play a key role in offspring survival. Furthermore, the variation in thrifty phenotype in F2 female mice mediates the effect of the maternal grandmothers’ strain on the maternal care of these F2 females. Since the strains of grandmothers have dramatic differences in maternal care, this mediation suggests maternal effects in which both maternal care and thrifty might be passed down over generations via epigenetic transmission. To our surprise, however, stress reactivity seemed to play no role in this relationship. A closer look at the results can provide a richer picture of our findings, and we discuss this below.

The phenotypes of maternal care that we measured here all loaded consistently well into a latent factor. Although expected, it is notable that measured phenotypes as distinct as the height of a nest and time spent licking/grooming share common variance. (In other words, a portion of the individual differences seen observed in those measures have the same cause.) The same was true for the latent factor of thrifty phenotype, where phenotypes as distinct as reduced weight of thrift organs and weight gain during early life seem in agreement. Unexpectedly, though, the variable Time Freezing in the Forced Swim test did not load into the latent phenotype of Stress Reactivity. The Forced Swim test is frequently used to measure behaviors related to motivation and depression, and those are commonly, but not necessarily linked to stress. In fact, some mouse studies have found either small or nonsignificant correlations between results in the Forced Swim test and anxiety-related behaviors measured in the Elevated Plus Maze [46,47]. Another mouse study found that antidepressants reduce immobility in the Forced Swim, but do not affect repetitive behaviors such as self-grooming [48]. In light of this evidence, it is plausible that the variation in the Forced Swim test that we found here indeed has little in common with the variation in stress reactivity captured by our tests of self-grooming, Elevated Plus Maze, and Open Field.

The confirmatory factor analyses showed that the covariate of litter size did not fit well in the model. This was unexpected, since litter size is a factor known to be related to maternal performance and offspring growth in mice [19]. Indeed, even in our data, Litter Size had a significant correlation with Nest Presence, Nest Height, Milk Ejection, and Survival Rate. It is possible that controlling for litter size in the initial measurement model reduced the common variance between our measured variables–which then resulted in a model with inadequate general fit. If this was the case, then our main structural model (which does not have litter size) might be slightly less robust and/or realistic than we imagine. On the other hand, litter size did have significant correlations to all the measured phenotypes behind Thrifty or Stress Reactivity. This could mean that litter size does not play a role in their relationship with maternal care, and was therefore irrelevant in our main model.

Regarding our main model, we found that higher levels of the thrifty phenotype led to lower maternal care. Additionally, when compared to an alternative model without Thrifty Phenotype, our results suggest that this latent variable is indeed important to the relationship between maternal origin (Maternal Grandmother) and maternal care. This idea is further supported in the main model by the existence of a partial mediation effect of Maternal Grandmother on Maternal Care via Thrifty Phenotype. The mediation suggests that maternal care in the maternal grandmother (the SM/J strain of good mothers vs. the LG/J strain of bad mothers) is passed down to mothers in F2 generations via metabolic changes in early life. Note that the different direction of parental crosses in our study (explained in Methods) may have played a role in that mediation. Individual differences in F1 and F2 mice may be due to genomic imprinting based on gender–such as the gene *Peg3* that we previously found in the same parental strains SM/J and LG/J, and is only expressed when coming from the paternal line [21]. Nonetheless, our results here show that the model with paternal grandmothers did not fit the data. Further, comparing the model with paternal grandmothers to our main model showed that the maternal line is indeed important for the overall relation between thrifty phenotype and maternal care. Maternal effects and genomic imprinting based on gender are two distinct phenomena that are incorrectly mixed together. Only the genes in the mother that cause the change in methylation are considered a maternal effect [49]. It is also worth mentioning that although F1 females have identical nuclear genomes, they do differ in their mitochondrial genome. The DNA from this organelle is only maternally inherited and has already been associated to aspects of maternal performance, such as milk production [50], and to thrifty phenotype [51]. Because of that, mitochondrial DNA could plausibly explain some of the Maternal Grandmother effect we found on Maternal Care and Thrifty Phenotype in F2.

One caveat to our findings regarding Thrifty is that this trait may be strongly related to size, which was the phenotype originally selected to create the SM/J and LG/J strains. These two inbred strains were selected for body size for more than 50 years (for history and details, see [24]), and the intercross of these strains showed a complex genetic background for obesity [52,53]. Hence, although these strains were not selected for maternal care or stress reactivity, they could have been selected indirectly towards both extremes of thrifty phenotypes. If this is the case, the relationship between thrifty phenotypes and maternal care that we found here could be merely a product of chance during the selection of the original strains. We do not think that this is the case. It is important to note that the three phenotypes we measured here as indicators of Thrifty are, at least at face value, quite different from total weight and size. A strain selected for bigger size as adults would not necessarily show faster weight gain early in life or lower weight of organs as adults. Instead of size, we believe that food deprivation in early life is behind the connection between poor maternal care and thrifty phenotype. The individuals of the current study were the same as the ones we found QTLs for nursing and nest building [17,20]. And as reported previously, the F2 females that did not nurse properly had offspring with increased risk of developing the thrifty phenotype [17]. The offspring of poor F2 mothers had lower birth weight followed by rapid catching up that led to weight overcompensation and fat deposition by the time of weaning. Also, in a series of unrelated studies in rats, researchers found that protein deprivation in early life led to permanent weight reduction of some organs such the liver, lungs, and heart relative to total body weight, while the brain was unaffected [54]. In fact, a review of the literature on animals and humans suggests that nutritional thrift has a differential impact on body parts according to their relation to short-term survival; with muscles, reproductive organs, and liver sacrificed first [55]. As we discussed above, during times of nutritional thrift, the developing organism seems to divert resources to energy (fat) accumulation instead of other functions. Based on this evidence, we believe that the effect of Thrifty is not merely fortuitously correlated with poor maternal care. Instead, it is more likely that the metabolic disorder is directly related to the maternal effects passed down from mother to offspring.

By compared the first proposed model to the model without Stress Reactivity, our results showed that this latent phenotype is not relevant. In addition, there is no effect of Maternal Grandmother on Stress Reactivity, suggesting further that there is no maternal effect related to stress reactivity–though this does not exclude genetic effects. These results are extremely surprising. In stressful situations, prolonged exposure to elevated levels of stress hormones can promote, among other effects, insulin resistance, abdominal fat deposition, increased risk of arterial damage, and heart disease [56,57]. Furthermore, since stress reactivity is quite beneficial for the “flight, fight, and foraging” responses, it is easy to imagine environments where damage from high levels of stress would be an acceptable trade-off under dire circumstances [16]. In rats, offspring of mothers with poor maternal care show decreased open-field exploration, and longer latencies to eat food in a novel environment; these differences appear related to increased receptor levels and mRNA expression of corticotropin-releasing factor [58,59]. Here, however, we only measured stress reactivity indirectly via behavioral tests. Although stress hormone levels like corticosterone are known to be correlated with the tests we used, the correlation is not perfect. We hope that future studies can help elucidate this relationship.

A last point must be made regarding the directionality of the relationship between Maternal Care and Thrifty Phenotype: Although our main model structures maternal care as an effect caused by thrifty phenotype, this might not be the case. An alternative model with changed directionality between these two latent phenotypes fitted the data well, and was statistically equivalent to our main model. Nonetheless, there is a strong case for our main model because our measures of weight gain occurred during the first days of development and had a high load on Thrifty Phenotype. The results of the directionality between maternal care and metabolic changes are of great theoretical interest. There is a heated debate in the literature on two different hypotheses on the relationship between maternal effects and investment (for an extensive review on the topic, see [60]). The first hypothesis, called the Predictive Adaptive hypothesis, expects thrifty phenotypes to develop as an adaptation from offspring. This hypothesis therefore expects that signals of these metabolic disorders will appear early in life and cause problems in future maternal care. The second hypothesis, called the Maternal Capital hypothesis, expects thrifty phenotype to develop due to an adaptation by the mother; according to this hypothesis, maternal care would change first due to environmental needs, and then metabolic disorders would occur in the offspring. In our setting here, signs of thrifty phenotype appeared very early on in both the F2 generation (as reported here) and in the F3 generation (as reported by Goes et al. [17]). Because of this, our fitted model matches the conditions expected from the scenario of Predictive Adaptive hypothesis, although our evidence is rather weak and indirect. It is worth mentioning that a recent study in macaques suggests a similar conclusion, where maternal care in an environment with low food and high stress led to an adaptation in offspring that resembles the thrifty phenotype [61].

To conclude, we believe that our results will greatly contribute to the foundation of future studies on the role of maternal effects, and, in particular, further help the mapping of genes for maternal performance and thrifty phenotype, as well as their combined effects across generations.
